# A Lower Dose of Infection Generates a Better Long-Term Immune Response against *Toxoplasma gondii*

**DOI:** 10.4049/immunohorizons.2300006

**Published:** 2023-02-22

**Authors:** Magali M. Moretto, Jie Chen, Morgan Meador, Jasmine Phan, Imtiaz A. Khan

**Affiliations:** * Department of Microbiology, Immunology, and Tropical Medicine, The George Washington University, Washington, DC; † Department of Medicine, The George Washington University, Washington, DC

## Abstract

*Toxoplasma gondii*, an obligate intracellular pathogen, induces a strong immune response in the infected host. In the encephalitis model of infection, long-term protective immunity is mediated by CD8 T cells, with the CD4 T cell population providing important help. Most of the immune studies have used a 10- to 20-cyst dose of *T. gondii*, which leads to T cell dysfunctionality during the late phase of chronic infection and increases the chances of reactivation. In the current study, we compared the immune response of mice orally infected with either 2 or 10 cysts of *T. gondii*. During the acute phase, we demonstrate that the lower dose of infection generates a reduced number of CD4 and CD8 T cells, but the frequency of functional CD4 or CD8 T cells is similar in animals infected with two different doses. However, Ag-experienced T cells (both CD4 and CD8) are better maintained in lower dose–infected mice at 8 wk postinfection, with an increase number functional cells that exhibit lower multiple inhibitory receptor expression. In addition to better long-term T cell immunity, animals infected with a lower dose display reduced inflammation manifested by lesser Ag-specific T cell and cytokine responses during the very early stage of the acute infection. Our studies suggest a previously unappreciated role of dose-dependent early programming/imprinting of the long-term CD4/CD8 T cell response during *T. gondii* infection. These observations point to the need for an in-depth analysis of how early events shape long-term immunity against this pathogen.

## Introduction

Considered one of the most successful human pathogens (1), *Toxoplasma gondii* is an obligate intracellular pathogen transmitted by ingestion of contaminated food and/or water. Toxoplasmosis is considered one of the neglected parasitic infections in the United States and is included in a group of five parasitic diseases that have been targeted by the Centers for Disease Control and Prevention for public health action (CDC). A recent metadata analysis in pregnant women revealed a global IgG seroprevalence of 32.9% with some regions presenting a prevalence of >45% (2). Furthermore, a new Australian study reported a seropositivity rate of >60% that increased significantly with age (3). In addition to pregnant women and newborn babies (4), the parasites can cause severe complications in immunocompromised individuals, especially those suffering from AIDS (5). HIV-infected individuals carrying latent toxoplasma infection develop encephalitis, and it is believed that the reactivation occurs during the advanced stage of the disease when CD4 T cells are severely depleted (6). The underlying cause for the reactivation of this chronic parasitic infection is a suboptimal level of the CD4 Th cell response, which compromises the primary effector CD8 T cells that are essential for keeping the chronic infection under control.

A murine model of *T. gondii* infection has demonstrated a synergistic role for both CD4 and CD8 T cells in controlling the infection and maintaining the chronicity (7). However, the importance of CD4 T cells is limited to safeguarding the functional CD8 T cell response, which prevents the reactivation of the disease (8). Several laboratories, including ours, have demonstrated the major role of effector CD8 T cells in the containment of toxoplasma infection (9–15). Studies from our group have established that in an encephalitis model of infection, CD8 T cells from mice carrying a latent toxoplasma infection exhibit an increased expression of inhibitory receptors, especially PD-1, and ultimately become dysfunctional (16). CD8 T cell dysfunctionality was attributed to the loss of adequate help from exhausted CD4 T cells (17). Despite the available information related to protective immunity against *T. gondii* infection, there is a paucity of data regarding the factors that shape the long-term CD8 T cell response. A study conducted with a viral infection has demonstrated that the programming of the CD8 T cell memory response is dependent on initial antigenic encounters and can be controlled by exposing the cells to low, intermediate, or high doses of infection (18). However, the host’s immune response to different doses of *T. gondii* has not been evaluated yet. Therefore, we set out to determine the host immune response to 2 versus 10 cysts of *T. gondii*, focusing especially on the long-term immunity during late chronic infection. Interestingly, we observed that a higher dose (HD) infection induces a stronger innate and adaptive immune response during the very early stage of the infection as compared with a lower dose (LD) challenge. Nevertheless, an LD challenge ultimately leads to a significantly higher CD8 T cell memory response in terms of both cell numbers and functionality at week 8 postinfection (p.i.), as well as decreased inhibitory receptor expression as compared with mice infected with an HD. To the best of our knowledge, these observations demonstrate, for the first time, that the dose of infection can be a factor in the development of optimal long-term CD8 T cell immunity during chronic toxoplasma infection.

## Materials and Methods

### Animals

All animal studies involved C57BL6/J mice (7 to 8 wk old) purchased from The Jackson Laboratory and were carried out in accordance with the Institutional Animal Care and Use Committee–approved guidelines at The George Washington University.

### Parasites and infection

Mice were infected orally with 2 or 10 cysts of the Me49 *T. gondii* strain prepared from the brain of chronically infected CBA/J mice (4–5 wk p.i.). *T. gondii* lysate Ag was generated from the RH strain maintained in vitro as previously described (9). When necessary, the clinical score was assessed weekly in a double-blind manner. The clinical score was defined as follows: 0, normal; 1, slightly ruffled fur; 2, swollen abdomen, ruffled fur; 3, arched back, swollen abdomen, ruffled fur; 4, slightly less active, arched back, swollen abdomen, ruffled fur; 5, low activity, labored breathing, eyes shut, arched back, swollen abdomen, ruffled fur. Mice with a clinical score >4 were immediately euthanized.

### *T.gondii* quantification

Parasite burden was assessed in half of the brain of infected animals as previously described (19). DNA was extracted from the tissues using a DNeasy kit (Qiagen) according to the manufacturer’s instructions. Amplification of 400 ng of purified tissue DNA was performed with primers specific for the B1 gene (35 tandem copies) (16). Known parasite equivalents were used to establish a standard curve.

### Lymphocyte preparation and flow cytometry

Splenocytes were prepared and stained as previously described. Abs used in the studies were as follows: BioLegend: CD8β (YTS156.7.7), CD4 (GK1.5), CD11a (M17/4), CD44 (IM7), KLRG1 (2F1/KLRG1), TIGIT (1G9), IFN-γ (XMG1.2), CD107a (1D4B), NK1.1 (PB136), CD11b (M1/70), Gr1 (RB6-8C5), CD80 (16-10A1); Thermo Fisher Scientific: CD49d (R1-2), LAG-3 (C9B7W), TIM3 (RMT3-23), TNF-α (MP6-X22), CD11c (N418), CD86 (GL-1). Live/Dead Aqua staining (Thermo Fisher Scientific) and fluorescence minus one were included in all experiments. Intracellular staining for cytokine production was performed after 20 h in vitro stimulation with 20 µg/ml *T. gondii* lysate in supplemented Iscove’s DMEM media. Protein transport inhibitor (Thermo Fisher Scientific) and labeled anti-CD107a were added to the cell suspension for the last 4 h of incubation. Stained cells were acquired with a FACSCelesta cytometer (BD Biosciences), and data were analyzed with FlowJo software. Data were systematically cleaned using the flowAI plugin, and events that deviated outside the statistical norm were excluded. The SPICE program provided by M. Roederer (National Institutes of Health, Bethesda, MD) was used for computing multiple markers.

Annexin V staining was performed as previously described (20). Briefly, splenocytes were isolated and rested for 4 h at 37°C prior to annexin V staining in accordance with the manufacturer’s recommended protocol (Thermo Fisher Scientific).

### ELISA

Detection of IgM and IgG2a in the serum of infected animals was performed according to the published protocol with modification (21). Briefly, plates were coated with 6 µg/ml *T. gondii* lysate, and serial dilution of serum was incubated for 1 h at room temperature. After incubation with HRP-conjugated secondary polyclonal anti-IgM or IgG2a (Thermo Fisher Scientific), tetramethylbenzidine substrate was added, and the titers for IgM and IgG2a were determined to be the inverse of the first dilution that was 2.5-fold higher than the absorbance at 450 nm of the negative control (PBS). IFN-γ and IL-10 detection in the serum was determined with the ELISA MAX Deluxe set (BioLegend) according to the manufacturer’s protocol.

### Cytokine mRNA detection by PCR

Real-time detection of IFN-γ, TNF-α, IL-10, and IL-12 was performed as previously described (20). RNA isolated with TRIzol (Invitrogen) was converted to cDNA using a high-capacity cDNA reverse transcription kit (Applied Biosystems) after contaminating DNA removal with a DNA-free kit (Ambion). Real-time PCR amplification was performed with CFX96 (Bio-Rad) for 40 cycles: 95°C for 45 s, 61.4°C for 50 s, and 72°C for 45 s. The sequences of the primers used are as follows: IL-12p40 forward, 5′-AGATGAAGGAGACAGAGGAG-3′, reverse, 5′-GGAAAAAGCCAACCAAGCAG-3′; IL-10 forward, 5′-GGTTGCCAAGCCTTATCGGA-3′, reverse, 5′-ACCTGCTCCACTGCCTTGCT-3′; IFN-γ forward, 5′-TGGCTCTGCAGGATTTTCATG-3′, reverse, 5′-TCAAGTGGCATAGATGTGGAAGAA-3′; TNF-α forward, 5′-TGGGAGTAGACAAGGTACAACCC-3′, reverse, 5′-CATCTTCTCAAAATTCGAGTGACAA-3′; RPL9 forward, 5′-TGAAGAAATCTGTGGGTCG-3′, reverse, 5′-GCACTACGGACATAGGAACTC-3′. Gene expression was normalized using RPL9 as an endogenous control.

### Statistical analysis

Statistical significance for percentages, absolute numbers, mean fluorescence intensity (MFI), and cytokine gene expression was evaluated using a Student *t* test. Statistical significance is indicated in the figures as follows: *p < 0.05, **p < 0.01, ***p < 0.001, and ****p < 0.001. Error bars presented in each graph represent the SD of the value between individual mice from each group. All computations were calculated using GraphPad Prism software.

## Results

### The initial inferior T cell response to a lower infective dose is better maintained during the chronic stage of infection

**FIGURE 1.**
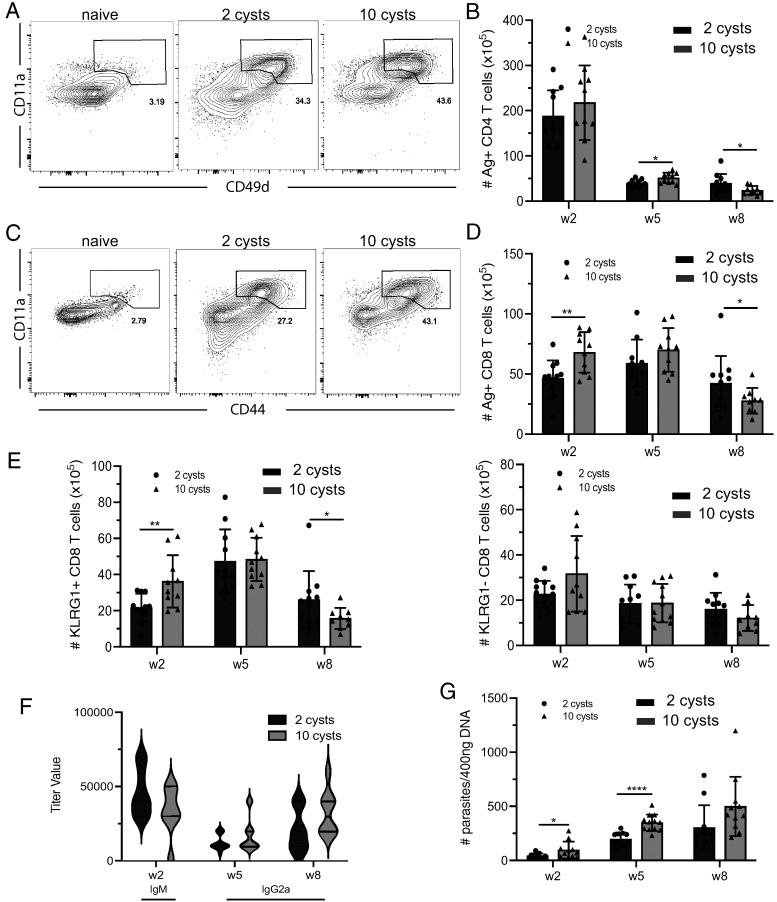
The initial inferior T cell response to a lower infective dose is better maintained during the chronic stage of infection. C57BL/6 mice were infected via the oral route with 2 or 10 cysts of *T. gondii*, and the immune response was evaluated at weeks 2, 5, and 8 p.i. (**A** and **C**) Ag^+^ CD4 T cells (gated on CD4 T cells) and Ag^+^ CD8 T cells (gated on CD8 T cells) were assessed using CD49d/CD11a (A) or CD44/CD11a (C) expression, respectively (week 2 p.i.). (**B** and **D**) The total numbers of Ag^+^ CD4 (B, CD49d^hi^CD11a^hi^) and CD8 (D, CD44^hi^CD11a^hi^) T cells were compared in the spleen of infected animals at weeks 2, 5, and 8 p.i. (**E**) The kinetic of KLRG1^+^ and KLRG1^−^ CD8 T cells (gated on Ag^+^ CD8 T cells) was evaluated at weeks 2, 5, and 8 p.i. (total number). (**F**) *Toxoplasma*-specific IgM (week 2 p.i.) and IgG2a (weeks 5 and 8 p.i.) were assessed in the serum by ELISA. (**G**) The parasite burden in the brain of LD- or HD-infected mice was measured by real-time PCR at different time points p.i. *p < 0.05, **p < 0.01, ****p < 0.001, by a Student *t* test. Experiments were performed at least three times (*n* = 4–6 mice/group), and graphs represent pooled data from three independent experiments (B–G).

Several laboratories, including ours, routinely use a murine model of *T. gondii* where an infective dose of 10–20 cysts of the type II strain is characterized by strong inflammation and a robust T cell immune response in susceptible C57BL/6 mice (22–26). In this study, we investigated the differences in Ag-specific CD4 and CD8 T cell immunity from mice infected with different doses of infection. For this purpose, animals were orally infected with 2 (LD) or 10 cysts (HD) of the Me49 strain. The Ag-specific/experienced (Ag^+^) CD4 and CD8 T cells from the spleen were evaluated at weeks 2, 5, and 8 p.i. unless otherwise specified. To ensure that only infected mice were included in our study, serum Ab titers and parasite burden in the brain were measured at 2, 5, and 8 wk p.i. Mice with an undetectable Ab titer and no measurable parasites were excluded from the study. In a mouse model using 10 cysts for infection, week 2 p.i. corresponds to the peak of the acute phase, and week 5 p.i. relates to the early chronic phase. Week 8 p.i. represents the late chronic phase of the immune response, and at this time point, dysfunctionality in the CD4 and CD8 T cell response has been reported (24, 25, 27, 28). As a tetramer strategy to identify Ag-specific CD4 or CD8 T cells in a toxoplasma model is currently very limited, we used a well-described surrogate marker approach, which has the added advantage of investigating polyclonal CD4 and CD8 T cell populations (29, 30). Therefore, as previously reported (17, 31), Ag^+^ CD4 cells were defined as CD49d^hi^CD11a^hi^, and Ag^+^ CD8 T cells were defined as CD44^hi^CD11a^hi^ (Fig. 1A and 1C, respectively). We observed that LD infection generated a lower Ag^+^ CD4 and CD8 T cell response at week 2 p.i. (Fig. 1B–D). Interestingly, the situation was reversed at 8 wk p.i., when the mice infected with an LD exhibited a higher Ag^+^ CD4 and CD8 response as compared with HD-infected mice (Fig. 1B–D). Ag^+^ CD8 T cells can be differentiated into effector and memory CD8 T cell populations based on their expression of KLRG1 (32). Although some memory CD8 T cells may express this receptor (33), based on our observations, effector CD8 T cells constituted most of the KLRG1^+^ subset in response to *T. gondii* infection (Supplemental Fig. 1). As shown in Fig. 1E, mice infected with an LD exhibited a significantly lower number of KLRG1^+^ (effector) CD8 T cells at 2 wk p.i., as compared with HD-infected mice. However, the KLRG1^+^ CD8 T cell response in LD-infected mice was better maintained at week 8 p.i., with a pattern comparable to the one observed in the total Ag^+^ CD8 T cell population. Although the differences in the KLRG1^−^ CD8 T cell (memory) response were not statistically significant between LD- and HD-infected mice, the trend was comparable to the KLRG1^+^ and Ag^+^ CD8 T cell population (Fig. 1E). Interestingly, no significant differences in the IgG2a response (which is known to be dependent on IFN-γ production [34]) and IgM levels between LD- or HD-infected mice were noted (Fig. 1F). To evaluate the ability of LD- and HD-infected mice to control the infection, the parasite burden was evaluated in the brain by measuring the expression of the B1 gene, which is conserved in both the tachyzoite and bradyzoite stages of the parasite (35). As expected, LD-infected mice exhibited a small number of parasites at week 2 p.i., which increased at week 5 p.i. but remained significantly lower than for HD-infected mice. However, the parasite burden was no longer different between LD- and HD-infected mice at week 8 p.i. (Fig. 1G). These findings point to definite dose-dependent variations in the immune response to acute *T. gondii* infection. While LD mice initially exhibited a reduced T cell response, it was significantly better maintained as compared with HD animals during the later stages of the infection.

### CD8 T cells from LD-infected mice exhibit increased apoptosis

**FIGURE 2.**
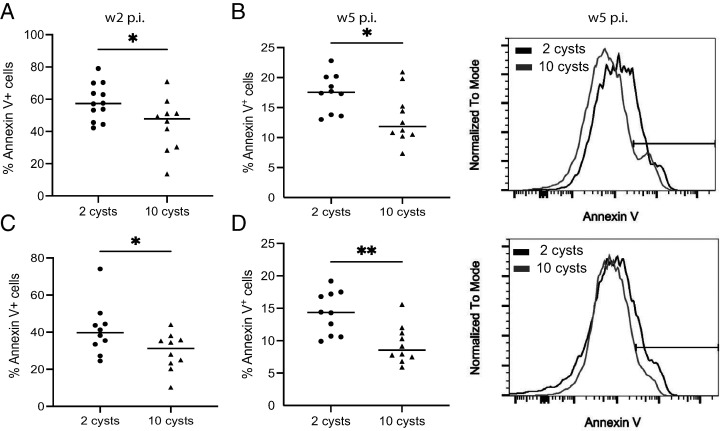
CD8 T cells from LD-infected mice exhibit increased apoptosis. Apoptotic splenic CD8 T cells from mice infected orally with 2 or 10 cysts of *T. gondii* were analyzed by flow cytometry. (**A** and **B**) The percentage of annexin V^+^ Ag^+^ CD8 T cells was compared between 2 and 10 cysts-infected mice at week 2 p.i. (A) and week 5 p.i. (B). (**C** and **D**) Similarly, the comparison between annexin V^+^ KLRG1^+^ CD8 T cells is presented at weeks 2 (C) and 5 (D) p.i. Histograms depict the gating strategy for annexin V^+^ Ag^+^ CD8 T cells (B) and KLRG1^+^ CD8 T cells (D). *p < 0.05, **p < 0.01, by a Student *t* test. Experiments were carried out at least three times (*n* = 4–6 mice/groups), and data are representative of three combined experiments.

CD8 T cells are the primary effector cells during *T. gondii* challenge and play a critical role in controlling the acute stage of the infection (14, 25, 28, 36, 37). It is well established that once the acute infection is resolved, a large proportion of the effector CD8 T cell population contracts by undergoing apoptosis whereas memory T cells are maintained to prevent reactivation of the infection or reinfection (38). Therefore, we compared the frequency of annexin V^+^ CD8 T cells from LD and HD mice at week 2 p.i. Interestingly, Ag^+^ (CD44^hi^CD11a^hi^) CD8 T cells from LD-infected mice were significantly more apoptotic at weeks 2 and 5 p.i. as compared with mice that received an HD infection (Fig. 2A, 2B). As expected, the effector (KLRG1^+^) CD8 T cell subset, which constituted most of the Ag^+^ CD8 T cells at these time points, behaved in a similar manner (Fig. 2C, 2D). Also, there was no significant difference in the frequency of the annexin V^+^ memory KLRG1^−^ CD8 T cell subset in LD- and HD-infected animals (data not shown). Our data demonstrate that a larger number of effector CD8 T cells are persisting in HD-infected mice to control the higher Ag load and therefore exhibit decreased apoptosis as compared with the cells from LD-infected animals.

### Polyfunctional CD8 T cells from LD-infected mice are better maintained during the chronic stage of infection

**FIGURE 3.**
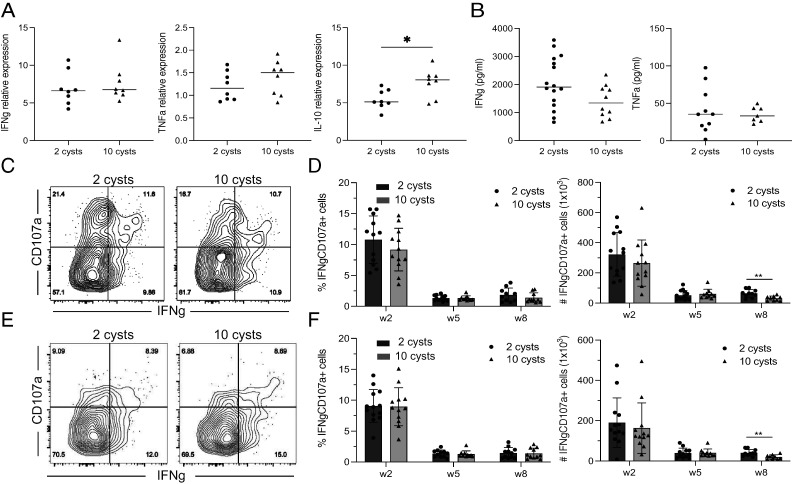
Polyfunctional CD8 T cells from LD-infected mice are better maintained during the chronic phase of infection. Mice were infected with 2 or 10 cysts of *T. gondii*. Cytokine production and cytotoxicity were assessed at weeks 2, 5, and 8 p.i. (**A**) IFN-γ, TNF-α, and IL-10 mRNA expression levels in the spleen were measured at week 2 p.i. by real-time PCR. (**B**) IFN-γ and TNF-α protein levels in the serum were also evaluated at the same time point by ELISA. (**C** and **E**) The functionality of Ag^+^ (C) and KLRG1^+^ (E) CD8 T cells was determined at week 2 p.i. by CD107a (cytotoxicity marker) detection on the surface of the cells and IFN-γ intracellular staining. (**D** and **F**) The frequency and total numbers of Ag^+^ (D) and KLRG1^+^ (F) CD8 T cells that were IFN-γ/CD107a^+^ were compared at weeks 2, 5, and 8 p.i. *p < 0.05, **p < 0.01, by a Student *t* test. Experiments were performed at least three times (*n* = 4–6 mice/groups), and graphs represent data pooled from two (A) or three independent experiments (B, D, and F).

IFN-γ is the hallmark of a strong immune response early after *T. gondii* infection (39). Unexpectedly, even though HD- and LD-infected mice displayed significant differences in the number of Ag^+^ CD4 and CD8 T cells at week 2 p.i., splenic IFN-γ and TNF-α mRNA expression was comparable in both groups (Fig. 3A). However, the mRNA level for IL-10, a cytokine important for offsetting the effect of high inflammation (40), was significantly higher in HD-infected compared with LD-infected animals (Fig. 3A). Similarly, serum levels of IFN-γ and TNF-α were not different between HD- and LD-infected mice (Fig. 3B). As the CD8 T cell response was associated with both IFN-γ secretion and cytotoxicity (16), the polyfunctional response in these cells was assayed by polychromatic flow cytometry. CD107a, a marker of degranulation, was used to measure the cytotoxic response. The percentage and total number of CD107a/IFN-γ^+^ cells in the Ag^+^ CD8 T cell population from HD- and LD-infected mice were comparable at weeks 2 and 5 p.i. despite HD-infected mice presenting significantly more Ag^+^ CD8 T cells at these time points (Fig. 3C, 3D). However, LD-infected mice exhibited a significant increase in the numbers of the polyfunctional CD8 T population at week 8 p.i., which correlated with a greater number of Ag^+^ CD8 T cells (Fig. 3D). Similarly, the number of IFN-γ/TNF-α^+^ Ag^+^ CD8 T cells in LD-infected animals was higher as compared with HD mice at this time point (data not shown). Effector KLRG1^+^ CD8 T cells, which constituted most Ag^+^ CD8 T cells at weeks 2 and 5 p.i., followed a similar pattern (Fig. 3E, 3F). Importantly, the number of polyfunctional KLRG1^−^ CD8 T cells (memory population) was also increased in LD-infected animals (Supplemental Fig. 2). These findings demonstrate that animals receiving an LD of infection develop a better long-term CD8 T cell response compared with mice challenged with an HD of *T. gondii*.

### Lower expression of inhibitory receptors by CD8 T cells from LD-infected mice

**FIGURE 4.**
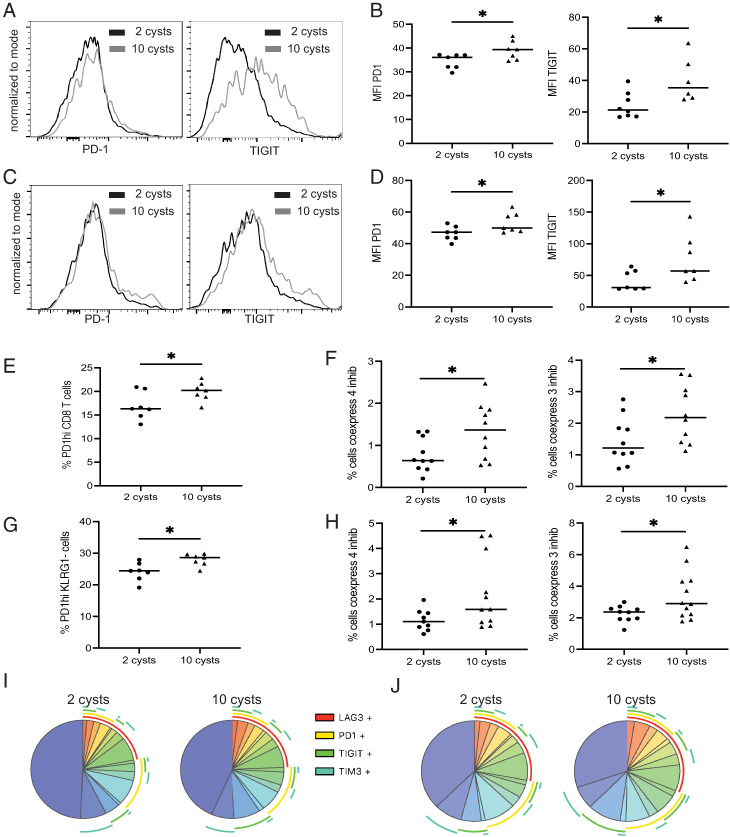
Lower expression of inhibitory receptors by CD8 T cells from LD-infected mice. C57BL/6 mice were infected via the oral route with 2 or 10 cysts of *T. gondii*, and the expression of several inhibitory receptors was evaluated at week 8 p.i. (**A**–**D**) Mean fluorescence intensity (MFI) for PD-1 and TIGIT was assessed for Ag^+^ (A and B) and KLRG1^−^ (C and D) CD8 T cells by flow cytometry. (**E**–**H**) Also, the frequency of PD-1^hi^ Ag^+^ and KLRG1^−^ CD8 T cells was determined at the same time point. The frequency of Ag^+^ and KLRG1^−^ CD8 T cells coexpressing a combination of four (PD-1, LAG-3, TIGIT, and TIM3) or three (PD-1, LAG-3, and TIGIT) inhibitory receptors was calculated. The frequency of inhibitory receptor (PD-1, LAG-3, TIGIT, and TIM3) coexpression by Ag^+^ and KLRG1^−^ CD8 T cells was visualized by SPICE analysis (**I** and **J**, respectively). *p < 0.05, by a Student *t* test. Experiments were carried out at least three times (*n* = 4–6 mice/group), and data were pooled from two (B, D, F, and G) or three independent experiments (F and H).

Previous studies from our laboratory showed that CD8 T cells from mice infected with 10 cysts of *T. gondii* exhibit an increased expression of PD-1 that results in loss of functionality during the chronic phase of infection (16, 27). Therefore, the expression of checkpoint inhibitors (PD-1, TIGIT, LAG-3, and TIM-3) was assessed at 8 wk p.i., when the immune response was declining and the parasite started to reactivate (27). As shown in Fig. 4A and 4B, Ag^+^ CD8 T cells from LD-infected mice displayed a significantly lower expression of PD-1 and TIGIT as compared with HD-infected animals. Moreover, akin to the Ag^+^ CD8 T cells, the memory (KLRG1^−^) CD8 T cell population from LD-infected mice had a significantly lower expression of the same inhibitory receptors (Fig. 4C, 4D). Also, LD-infected mice presented a reduced frequency of PD-1^hi^ Ag^+^ as well as memory CD8 T cells as compared with HD-infected mice (Fig. 4E and 4G, respectively). Furthermore, SPICE analysis demonstrated that Ag^+^ and memory CD8 T cells from LD-infected mice exhibited a significantly lower coexpression of multiple receptors (both three and four inhibitory receptors) as compared with the cells from HD-infected mice (Fig. 4F, 4H–J). These observations demonstrate that LD infection elicits lower expression of multiple inhibitory receptors, which most likely explains the better maintenance of Ag^+^ CD8 T cells during the late chronic infection.

### LD-infected mice display more CD4 T cells with lower inhibitory receptors and better functionality during chronic infection

**FIGURE 5.**
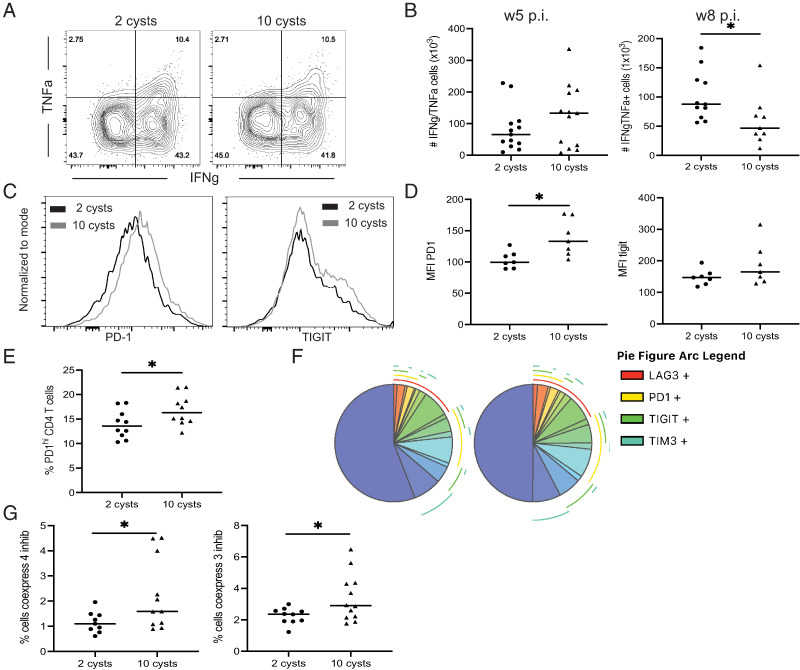
LD-infected mice display more CD4 T cells with lower inhibitory receptors and better functionality during chronic infection. Mice were infected with 2 or 10 cysts of *T. gondii*. (**A**) At week 2 p.i., Ag^+^ CD4 T cell functionality was assessed by measuring IFN-γ and TNF-α by intracellular staining. (**B**) The total number of IFN-γ/TNF-α^+^ Ag^+^ CD4 T cells in 2 and 10 cysts-infected mice were compared at weeks 5 and 8 p.i. (**C**–**E**) PD-1/TIGIT MFI (C and D) and the frequency of PD-1^hi^ (E) Ag^+^ CD4 were analyzed at week 8 p.i. (**F**) SPICE analysis of Ag^+^ CD4 T cells displays the frequency of Ag^+^ CD4 T cells coexpressing up to four inhibitory receptors (PD-1, LAG-3, TIGIT, and TIM3) at week 8 p.i. (**G**) Frequencies of Ag^+^ CD4 T cells from 2 or 10 cysts-infected mice coexpressing three (PD-1, LAG-3, and TIGIT) or four (PD-1, LAG-3, TIGIT, and TIM3) inhibitory receptors were compared at week 8 p.i. *p < 0.05, by a Student *t* test. Experiments were performed at least three times (*n* = 4–6 mice/group), and graphs represent data pooled from two (D) or three independent experiments (B, E, and G).

As stated above, although CD8 T cells are the primary effectors during *T. gondii* infection, the CD4 T cell population plays an important synergistic role (7, 8). Previous studies from our laboratory have reported that CD8 T cell exhaustion observed during chronic toxoplasmosis is a consequence of a dysfunctional CD4 T cell response (17). Even though Ag^+^ CD4 T cells from LD-infected mice exhibited a similar polyfunctional response at 2 wk p.i. (Fig. 5A), the number of Ag^+^ CD4 T cells that retained the ability to produce both IFN-γ and TNF-α at week 8 p.i. was significantly greater in LD-infected mice as compared with animals that received HD (Fig. 5B). As observed with CD8 T cells, this difference in the number of the IFN-γ/TNF-α^+^ CD4 T cell population at week 8 p.i. correlates with the differential expression of PD-1 reminiscent of the pattern observed in Ag^+^ CD8 T cells (Fig. 5C, 5D). As expected, Ag^+^ CD4 T cells from HD-infected mice were also comprised of a higher percentage of PD-1^hi^ cells (Fig. 5E). Furthermore, SPICE analysis demonstrated that the coexpression of multiple inhibitory receptors was also significantly lower in Ag^+^ CD4 T cells from mice infected with an LD of toxoplasma cysts (Fig. 5F–G). Ultimately, our data demonstrate that infection with LD generates an Ag^+^ CD4 T cell immunity that displays a less exhausted status associated with stronger functional fitness and most likely provides better help to CD8 T cells.

### HD-infected mice exhibit a stronger T cell immune response very early p.i.

**FIGURE 6.**
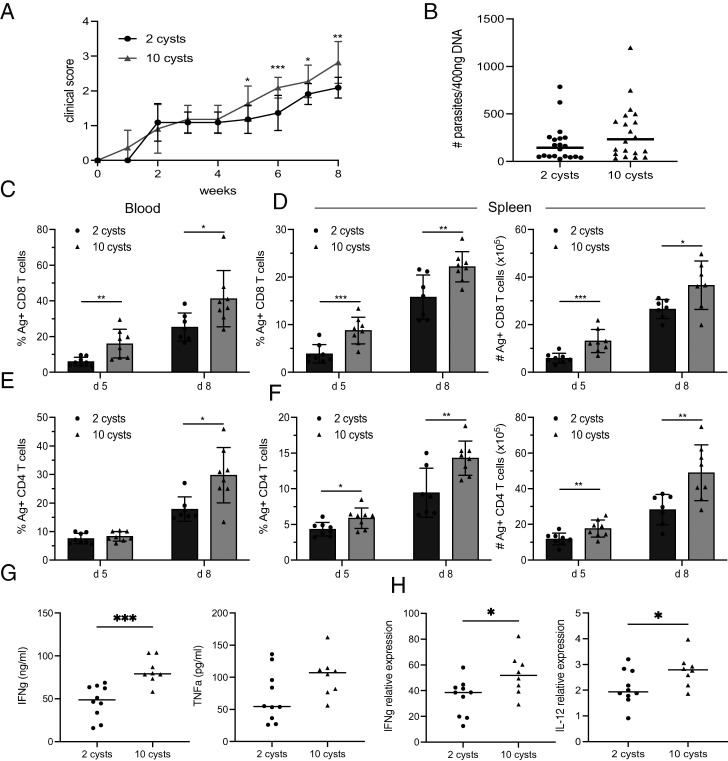
HD-infected mice exhibit a stronger T cell immune response very early p.i. Mice were infected with 2 or 10 cysts of *T. gondii*. (**A**) Double-blind clinical scoring was recorded for each infected mouse at different time points after infection with an LD or HD dose of T. *gondii*. (**B**) At week 8 p.i., the parasite burden was assessed by real-time PCR in the brain of 2 or 10 cysts-infected mice (five experiments with four to six mice/group pooled). (**C** and **E**) Frequency of Ag^+^ CD8 (C) and CD4 (E) T cells was assessed in the blood of infected mice at day 5 and 8 p.i. (**D** and **F**) Similarly, the frequency and total number of both subsets were measured in the spleen at the same time points. (**G**) IFN-γ and TNF-α levels were determined in the serum at day 8 p.i. by ELISA. (**H**) mRNA expression for IFN-γ and IL-12 was also measured in the spleen by real-time PCR at day 8 p.i. *p < 0.05, **p < 0.01, ***p < 0.001, by a Student *t* test. Experiments were carried out twice (*n* = 4–6 mice/group), and data were pooled from two (C–H), three (B), or five experiments (A).

The HD-infected mice displayed a significantly higher parasite burden, especially at week 5 p.i. (Fig. 1G), when the animals started to manifest early signs of toxoplasmosis (ruffled fur, hunch back) (Fig. 6A). As expected, HD-infected mice continued to exhibit an elevated clinical score at weeks 7–8 p.i. when the number of functional CD4 and CD8 T cells was decreased. However, at this time point, both groups presented a similar parasite burden (Fig. 1G) even when including large numbers of mice from multiple experiments (Fig. 6B, *n* = 20). As the role of early immune events in limiting *T. gondii* infection and shaping adaptive immunity is well documented, Ag^+^ CD4 and CD8 T cells were evaluated at day 5 and 8 p.i. Given that uninfected mice could not be excluded by measuring the Ab response or the parasite burden so early after infection, mice from either group that were consistently diverging from the average and/or were not different from naive mice were excluded from the analysis. Interestingly, we detected both Ag^+^ CD4 and CD8 T cells as early as day 5 p.i. in the spleen and blood of infected animals. However, KLRG1 expression was not observed at these early time points. Conspicuously, HD infection generated a more robust T cell response in the blood and spleen starting as early as days 5 and 8 p.i., with a significantly greater percentage of Ag^+^ CD8 (Fig. 6C, 6D) and CD4 (Fig. 6E, 6F) T cells as compared with LD-challenged animals. As expected, this robust T cell response was associated with a highly significant increase in levels of IFN-γ detected in the serum from the HD-infected mice at day 8 p.i. Although not statistically significant, an increase in TNF-α levels was also observed in these animals (Fig. 6E, 6F). Likewise, the mRNA expression for both IFN-γ and IL-12, a cytokine known to be critical for driving a protective immune response against *T. gondii* (10), was higher in the spleen of HD-infected mice (Fig. 6G, 6H). These observations demonstrate that the scale of the very early CD4 and CD8 T cell response is directly associated with the dose of infection.

**FIGURE 7.**
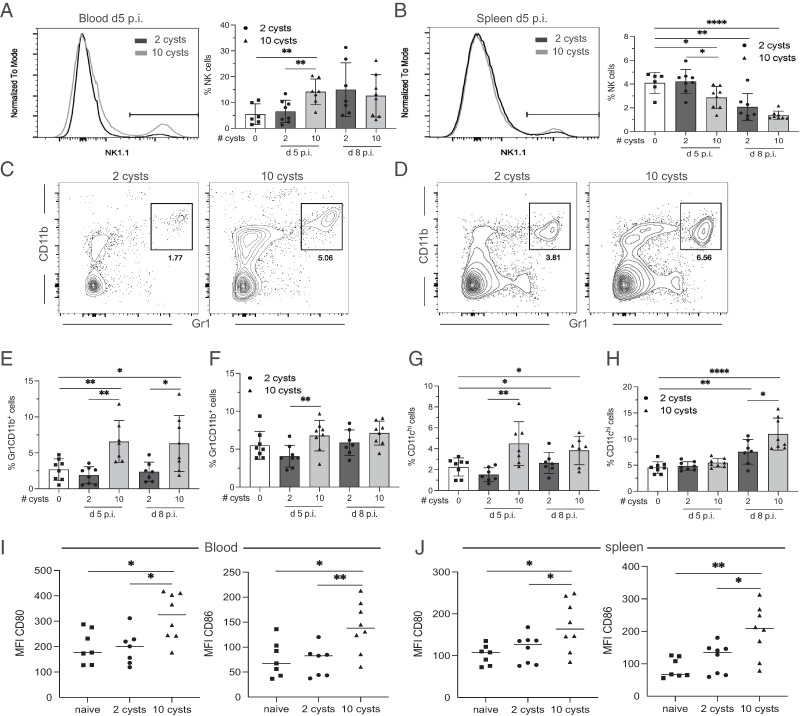
HD infection generates a stronger innate immune response. Mice were infected with 2 or 10 cysts of *T. gondii* and the innate immune response was assessed by comparing different cell subsets. (**A** and **B**) NK cells from infected mice were evaluated in the blood (A) and spleen (B) at days 5 and 8 p.i. by flow cytometry. (**C** and **D**) Similarly, Gr1/CD11b^+^ cells were analyzed in the blood (C) and spleen (D) of infected mice at day 5 p.i. (**E** and **F**) The frequency of Gr1/CD11b^+^ cells was also examined at days 5 and 8 p.i. in the blood (E) and spleen (F). (**G** and **H**) Likewise, the frequency of DCs (CD11c^hi^) was evaluated in the blood (G) and spleen (H) of infected mice at days 5 and 8 p.i. (**I** and **J**) The MFI of CD80 and CD86 expressed by DC (CD11c^hi^) from the blood (I) and spleen (J) of these mice was also assessed at day 5 p.i. *p < 0.05, **p < 0.01, ****p < 0.0001, by a Student *t* test. Experiments were performed twice (*n* = 4–6 mice/group), and graphs represent data pooled from two independent experiments.

Innate immunity provides an important first line of defense against *T. gondii* that is critical to control the rapidly growing infection (41). NK cells and neutrophils can recognize *T. gondii* and produce large amounts of IFN-γ, a cytokine essential for controlling the infection (42, 43). Crucial IFN-γ production by NK cells after *T. gondii* infection is IL-12–dependent (44). In contrast, neutrophils can produce both IFN-γ and IL-12 (45). Moreover, *T. gondii* infection induces neutrophil extracellular traps that can effectively kill parasites (46). As expected, the frequency of NK cells was significantly elevated in the blood of HD-infected mice at day 5 p.i. as compared with naive animals (Fig. 7A). Surprisingly, the NK cell percentage was not increased in the blood of LD mice (Fig. 7A). However, the frequency of NK cells decreased in the spleen of both HD and LD mice at day 8 p.i. (Fig. 7B). NK cells were possibly remaining longer in the blood of HD-infected animals to control the larger number of parasites still circulating and disseminating to the different tissues. Conversely, Gr1^+^CD11b^+^ cells, which comprise monocytes and neutrophils, were significantly more predominant in the blood and spleen of the HD-infected animals at day 5 p.i. (Fig. 7C–F). Similar to NK cell frequency, the percentage of Gr1^+^CD11b^+^ cells in LD-infected mice was not different from that of naive mice (Fig. 7C–F). Dendritic cells (DCs) are another important component of the innate immune response. In addition to being a central source of IL-12 (47), the role of DC (CD11c^hi^) as primary APCs is well established (48). HD-infected mice displayed a significant increase in the percentage of CD11c^hi^ cells in the blood as early as day 5 p.i. and in the spleen at day 8 p.i. (Fig. 7G, 7H). Interestingly, the increase in the percentage of DCs in LD mice was detected only at day 8 p.i. (Fig. 7G, 7H). Importantly, both blood and splenic DCs from HD-infected mice exhibited a significantly higher expression of costimulatory molecules CD80 and CD86, which are important in facilitating Ag presentation (49), at both time points tested (Fig. 7I, 7J). Overall, these findings suggest a stronger innate response in HD-infected animals that is likely necessary to limit the dissemination of infection. Conversely, weaker innate immunity in LD-infected mice could be linked to the decreased T cell response during the initial stage of infection (Fig. 6C–F).

## Discussion

*T. gondii* infection induces a strong innate and adaptive immune response in immunocompetent hosts (50), and the latter is dependent on both CD4 and CD8 T cells for keeping the chronic infection under control. Although CD8 T cells play a major effector role, CD4 T cell help is essential for maintaining their functionality (7, 8). Previous studies from our laboratory have reported that in an encephalitis model of infection, CD4 T cell dysfunction leads to CD8 T cell loss of function during the late stages of chronic infection (17). However, most of these studies were carried out with an infective dose of 10 cysts or higher. Although the effects of diverse host factors such as age (51) and sex (52) on the immune response to *T. gondii* have been considered, the role of Ag dose had not been evaluated. In the current study, we report that LD infection induces better long-term CD4 and CD8 T cell immunity, which is associated with fewer signs of exhaustion and better function.

Although an LD infection initially induces a significantly lower number of Ag^+^ CD8 T cells as compared with an HD infection (2 wk p.i.), surprisingly, there is no difference in the number or frequency of functional cells within this population (Fig. 3). Understandably, the number of parasites in LD-infected mice is significantly lower at the same time point. Even though our data demonstrate that LD-infected mice have a smaller number of Ag^+^ CD8 T cells during the acute phase, one of the most notable features of our study is the significantly higher number of Ag-specific CD8 T cells observed in LD-infected mice at 8 wk p.i., as compared with HD-challenged animals. Interestingly, the parasite load correlated initially with the dose of infection, but by week 8 p.i., animals from both groups presented a similar parasite burden. Additionally, the LD-infected animals started to be significantly sicker, based on their clinical score, at 5–6 wk p.i. and continued to be so until 8 wk p.i. The increased clinical score in the HD-infected mice, despite no differences in parasite load, may be attributed to the process of early reactivation that may not be detectable at this stage. This statement is based on our previous findings that demonstrated reactivation of the latent infection at 10–13 wk p.i. in mice challenged with an HD (16). The long-term maintenance of CD8 T cells has been reported to be important for controlling *T. gondii* reactivation (9), and the higher number of functional CD8 T cells observed in LD-infected mice most likely can be attributed to better CD4 T cell help, as these animals present a greater number of functional cells at 8 wk p.i.

Interestingly, the CD8 T cell response both in blood and spleen in HD mice as compared with LD animals was significantly increased as early as 5 d p.i., and the CD4 T cell response in the spleen followed a similar pattern. These findings are novel in the sense that, to the best of our knowledge, such an early adaptive immune response has not been previously reported after oral infection with *T. gondii*. However, the Ag^+^ CD8 T cell response seemed to wean faster and was comparable in both groups at week 5 p.i. Therefore, the number of functional CD8 T cells in LD-infected animals seemed to be better maintained, thus providing a stronger long-term immunity as compared with HD-infected mice. These findings indicate that an LD of infection can trigger the development of a more robust long-term immunity needed for the control of latent infection.

In chronic diseases such as cancer and several viral infections, the increased levels of inhibitory receptors on CD8 T cells lead to exhaustion and severely compromised protective immunity (53, 54). In recent years, T cell exhaustion has also been reported in parasitic diseases such as murine and human malaria and visceral leishmaniasis (55–57). Furthermore, we have reported the loss of CD8 T cell functionality during chronic toxoplasmosis caused by increased expression of checkpoint inhibitors, especially PD-1 (16, 24). In the current study, significantly lower expression of PD-1 was observed in Ag^+^ CD8 T cells from LD-infected animals. Moreover, the CD8 T cell population from these animals exhibited lower coexpression of multiple receptors (three or four inhibitory receptors) as compared with animals challenged with HD. These observations are important, as a stronger long-term CD8 T cell immunity (8 wk p.i.) in LD-infected mice may be due to lower exhaustion, which enables them to better maintain their functional response. The important observation made in our studies indicates that LD infection can induce better long-term CD4 and CD8 T cell immunity against *T. gondii* infection.

The importance of CD4 T cells in the protective immune response against *T. gondii* infection is well documented (7, 8, 17). Studies from our laboratory have demonstrated that although the CD8 T cell response against the parasite can be initiated without CD4 T cell help, it cannot be maintained (8). We have also reported that CD8 T cell dysfunctionality during chronic infection is a consequence of CD4 exhaustion (17). Taken together, these data support our findings that demonstrate a significantly higher and functionally improved CD4 T cell response in LD-infected animals at 8 wk p.i. Moreover, the cells from LD-infected mice also exhibited lower PD-1 and multiple inhibitory receptor expression as compared with the population in HD-challenged animals. Thus, as stated above, it is likely that an improved Ag^+^ CD4 T cell response in LD-infected animals can provide stronger help to CD8 T cells necessary for long-term protection against chronic infection.

Initially thought to be compartmentalized, innate and adaptive responses have been shown to form a very efficient network with a strong link between the two arms of the immune response. Neutrophils are the first cells to be recruited to the site of infection to help control the dissemination by producing reactive oxygen species and cytokines including IFN-γ and IL-12 (58, 59). Similarly, NK cells are a critical part of the innate immune response after *T. gondii* infection, and depletion of NK cells leads to an increase in parasite burden and higher mortality rate (44, 60). Furthermore, tachyzoite-infected human and mouse neutrophils release neutrophil extracellular traps, which kill the parasites and therefore limit their ability to disseminate (61). NK cells, along with CD4 T cells, are a critical source of IFN-γ during *T. gondii* infection (62). These cells can provide essential help to CD8 T cells, especially in the absence of a CD4 T cell population (8). As expected, the innate immune response in HD-infected mice presented significantly increased Gr1^+^CD11b^+^ and NK responses in the blood in comparison with LD-infected mice. Possibly, the most important interaction between innate and adaptive immune responses is the dependence of the T cell response on IL-12, a cytokine known for Th1 polarization (63) and driving a strong CD8 T cell effector response (64). IL-12 is indispensable for IFN-γ production during both the acute and chronic stages of *T. gondii* infection (65, 66). Understandably, early after infection, expression of both IFN-γ and IL-12 was significantly increased in HD-infected animals as compared with mice that received an LD of *T. gondii*. As IL-12 is mainly produced by DCs during *T. gondii* infection (67), it is not surprising that the increase in the IL-12 message is correlated with a higher DC response in the tissues (blood and spleen) of HD-infected mice in comparison with LD mice. Moreover, expression of costimulatory molecules (CD80 and CD86) that are known to result in better Ag presentation was also increased in DCs from HD-infected mice, leading to an improved T cell response (68). As expected, the scope of the innate immune response, which is known to be critical for controlling acute infection (41, 50), was correlated with the initial dose of infection, and mice that received the HD of *T. gondii* developed a stronger initial immune response. Moreover, LD-infected mice exhibited an overall delayed innate immune response, which could be responsible for lower inflammation and initial T cell immunity.

Thus, the ability of HD-infected animals to initially control the acute infection and survive the challenge may be a direct consequence of not only an early adaptive but also a stronger innate response. This could be attributed to the inflammatory cytokines that are comparatively increased very early in HD-infected animals, especially IFN-γ, a cytokine known to play a pivotal role in protection against acute infection (39). Although type I and II IFNs are well recognized for their immune-stimulatory activities (69), they also play an important role in the suppression of immune response against tumors (70). Although IFN-γ can directly increase the abundance of CD8 T cells during viral infection (71), it also affects the CD8 memory response by suppressing the expression of IL-7R (72), which is known to be important for the survival of these cells (73–75). Similarly, in a recent study with a tumor model, IFN-mediated activation of transcription factor IRF-2 has been reported to suppress the immune response (76). This study demonstrated that, as compared with LD infection, HD-challenged mice developed a stronger inflammation (IFN-γ, IL-12) during the earlier stages of infection needed to control the higher parasite dose. How increased IFN-γ levels in an HD model shape long-term CD8 T cell immunity during the very early stages of the infection is an important area of investigation for future studies.

Although, as pointed out in a recent report (77), chronically stimulated CD8 T cells share common phenotypic characteristics, including transcriptional and epigenetic programming, a better understanding of CD8 T cells in each disease is needed to provide novel individual strategies for their cure. We predict that better programming of T cells during the initial stages of infection in LD-infected mice results in stronger long-term CD8 T cell immunity. An in-depth transcriptomic analysis of these cells needs to be performed to identify factors that lead to superior CD4/CD8 memory during *T. gondii* infection. Future studies in our laboratory will be able to address these interesting questions that have important therapeutic implications.

## Supplementary Material

Supplemental Figures 1 (PDF)
