# Supplementary material for: A Lower Dose of Infection Generates a Better Long-Term Immune Response against Toxoplasma gondii
Source: Immunohorizons. 2023 Feb 22;7(2):177–90. doi: 10.4049/immunohorizons.2300006 (PMC10563383; doi:10.4049/immunohorizons.2300006)
Supplement: Supplemental Figures 1 (PDF) [file IH_2300006_Supplemental_1.pdf]

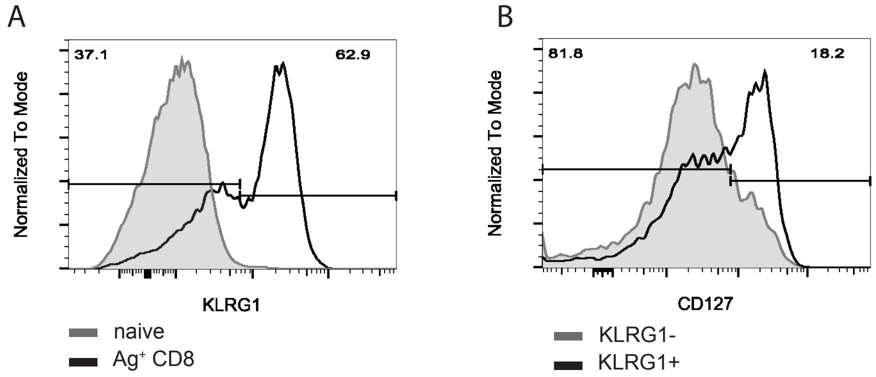

Supplementary figure 1: KLRG1^{+} Ag^{+} CD8 T cells are predominantly comprised of effector T cells.

Mice infected with 10 cysts of *T. gondii* were sacrificed at week 2 p.i. and splenic Ag^{+} CD8 T cells were evaluated for KLRG1 expression (A). CD127 expression by KLRG1^{+} CD8 T cells (gated on Ag^{+} population) was assessed (B). The experiment was performed three times and the data is representative of one experiment.

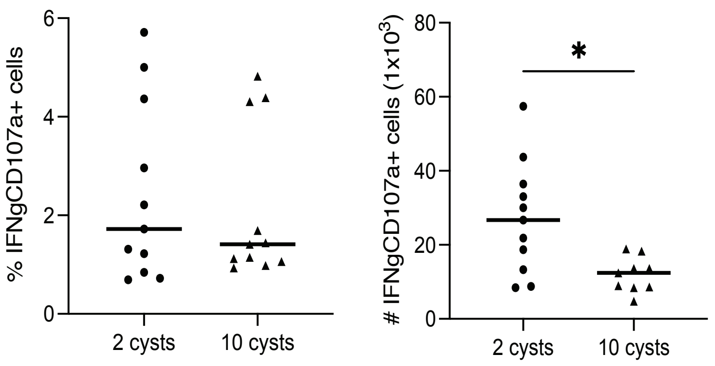

Supplementary figure 2: low dose infected mice display a larger number of functional memory ($KLRG1^-$) CD8 T cells during the chronic phase of infection.

Mice were infected with 2 or 10 cysts of *T. gondii*. At 8 weeks post-infection, animals were euthanized and splenocytes were evaluated for polyfunctionality after overnight stimulation with toxoplasma antigen extract. Data are presented as the frequency and total number of $KLRG1^-$ CD8 T cells (gated on Ag^+ ($CD44^{hi}CD11a^{hi}$) CD8 T cells) that co-expressed both IFNγ and CD107a. Statistical significance is presented as *= $p<0.05$. The experiment was performed three times and the graphs represent data from three pooled experiments.
